# The effects of PRRS on the environmental impact of pig production: a life cycle assessment study

**DOI:** 10.3389/fvets.2025.1625581

**Published:** 2025-12-11

**Authors:** Greg J. Thoma, Lucina Galina Pantoja, Daniel C. L. Linhares, Ana Paula Serafini Poeta Silva, Lindsay Case, Pieter W. Knap

**Affiliations:** 1 Resilience Services PLLC, Denver, CO, United States; 2 Genus-PIC, Hendersonville, TN, United States; 3 College of Veterinary Medicine, Iowa State University, Ames, IA, United States; 4 Genus-PIC, Isernhagen, Germany

**Keywords:** LCA, PRRS, pig, environmental impact, climate change, global warming

## Abstract

The Porcine Reproductive and Respiratory Syndrome (PRRS) is a viral pig disease that increases mortality rates and reduces (re)production performance. This worsens a farm’s whole-enterprise feed conversion ratio, which increases its greenhouse gas emission. PRRSV elimination should then result in mitigation of global warming. This study quantifies such mitigation of global warming and of other environmental impact categories such as eutrophication and land use by Life Cycle Assessment. The software package openLCA converted material and energy flows to impact categories of frameworks ReCiPe-2016, PEF-3.1, and IPCC-2021. Flows came from data recorded on 173 PRRS outbreaks in 113 sow farms (1.3 million sows), and on 5,650 all-in-all-out groups of wean-to-finish pigs in 63 farms (26 million pigs). We find that the environmental impact of PRRSV-negative North American farms is 4–6% lower than of the North American industry average (a mix of PRRSV-negative and PRRSV-positive farms), and 9–17% lower than of PRRSV-positive farms.

## 1 Introduction

The Porcine Reproductive and Respiratory Syndrome (PRRS) is a viral disease that affects domestic pigs and other Suoid species. The disease was first reported in 1987 in USA and since then, the virus (PRRSV: *Betaarterivirus suid*) has spread all over the pig-producing world; only a handful of countries in South America, Oceania and Europe (together holding about 7% of the global pig population) are currently PRRS-free. At the sow level, the clinical signs of PRRS include increased rates of sow mortality, abortion, and piglet mortality (prenatal, perinatal and preweaning) and reduced conception rates; in grower-finisher pigs the clinical signs include increased postweaning mortality and reduced feed intake, growth rate, and feed efficiency (1) (p. 693–694). Coinfections with other pathogens are common and can intensify the disease process (2) and also lead to considerably increased usage of antibiotics (3). All this seriously compromises animal health and welfare, and also the mental wellbeing of farm staff and the public perception of pig production (4); these issues are very difficult to quantify, but the economic impact has been studied in much detail.

Boeters et al. (5) reviewed 15 studies published from 1994 to 2022 that reported on the financial impact of endemic PRRS in China, Korea, Vietnam, USA, Ireland, Germany, the Netherlands, Italy and Poland; when feed costs were included in the analyses, the financial losses ranged from 3 to 9 EUR per nursery pig, from 3 to 15 EUR per grower-finisher pig, and from 200 to 300 EUR per sow per year. More recently, Meléndez-Arce et al. (6) estimated even higher losses for pig farms in Costa Rica, and Osemeke et al. (7) estimated losses in USA in 2016–2020 that were 80% higher than a decade earlier.

These detrimental effects of PRRS on survival and production traits translate to a detrimental effect on the environmental impact of pig production, mediated by a farm’s whole-enterprise feed conversion ratio (FCR), i.e., the amount of feed required to produce a particular quantity of slaughter pig. FCR is strongly correlated to nitrogen excretion (8–13), which is the main contributor to ammonia and greenhouse gas (GHG) emission from monogastric mammals. We may then expect a successful PRRSV elimination program to result in a correlated reduction of those emissions: “mitigation as a co-benefit to improved production efficiency” (14). Kyriazakis et al. (15) discuss this topic in more general terms and in much useful detail. Indeed, PRRSV infection of unvaccinated pigs significantly increased their manure emissions of CO_2_ and N_2_O, but not of NH_3_, CH_4_, and H_2_S (16). Likewise, Capper (17) modeled the impact of various livestock diseases on GHG emission and found that reduction of the prevalence of PRRS from 60 to 10%, foot and mouth disease in beef cattle from 45 to 5%, or avian infectious bronchitis in poultry from 75 to 20% would reduce GHG intensities by 22, 9, and 11%, respectively.

Such effects can be expected not only for GHG emission with its effect on global warming but also for other environmental impact categories such as eutrophication, acidification, and land use. Our aim is to quantify the effect of PRRSV elimination on such environmental impact categories via Life Cycle Assessment (18, 19), currently the most effective way to quantify such impacts.

## 2 Materials and methods

### 2.1 Data overview

The data used in this study were compiled by Iowa State University’s department of Field Epidemiology in the framework of its PRRS Outbreak Management Program (POMP;^1^ accessed on 09 November 2025) for sow farm data and its Predictors of Swine Performance (PROSPER;^2^ accessed on 09 November 2025) for wean-to-finish farm data.

For sow farms, the data covered 173 PRRS outbreaks distributed across 113 sow farms within six production systems in USA (about 1.3 million sows; in this context, a “production system” is an organization or company that controls several farms and imposes more or less uniform operating procedures for animal management, housing, nutrition and/or healthcare). In brief, the sow farm eligibility criteria to be included in POMP are (i) the farm experienced a PRRS outbreak as defined by the herd veterinarian; (ii) the farm and its veterinarian had a plan to eliminate the virus; (iii) the farm collected biological samples over the course of outbreak to determine the PRRSV infection phases according to the American Association of Swine Veterinarians (AASV) PRRSV status [(20, 21); see section 2.2]; (iv) the farm shared diagnostic results of weekly routine PCR tests for PRRSV RNA; (v) the farm completed the POMP survey with information about the management plan following the outbreak; (vi) the farm shared weekly production data records 52 weeks before and after the outbreak.

Our study used this data to determine sow farm infection phases. To focus on clinical PRRS outbreaks, those with time-to-baseline-production (the number of weeks post-outbreak to recover weaned piglet productivity) less than 5 weeks (typically associated with mild or vaccine virus strains), or with concurrent Porcine Epidemic Diarrhea (PED) outbreaks, had been removed from the data (22). Traits included in the analysis are total sow inventory, sow mortality rate, litter size (total number born), numbers stillborn including mummies (total born minus born alive), preweaning mortality rate, and piglets weaned per sow per year.

For wean-to-finish (WtF) farms, the data cover 5,650 closeouts (i.e., all-in-all-out groups of wean-to-finish pigs housed together and marketed together), distributed across 63 farms within two production systems in USA (about 26 million finisher pigs). The automated PROSPER algorithms integrated information from the sow farm (PRRSV infection statuses based on the AASV classification) to the WtF phase, including diagnostic testing reports during this latter phase. Thus, the PCR-based infection status of the animals at WtF entry is known. Closeouts that are PCR-negative at entry may experience an outbreak sometime later, as diagnosed by the farm’s veterinarian. Closeouts with concurrent PED outbreaks had been removed from the data. Traits included in the analysis are age at entry and at exit (leading to days-on-feed), entry weight, weight at exit (market weight), postweaning mortality rate, growth rate, and feed conversion ratio adjusted for days-on-feed.

### 2.2 Infection phases

We follow a compartmental epidemiological model (CEM) approach, where a population progresses from one compartment to the next (classically: from susceptible via infectious to recovered) (23, 24). CEM was developed for the estimation of epidemiological parameters such as the basic reproduction number R_0_ of the pathogen of interest, but our study aims to compare the relevant compartments in terms of their production performance traits and, from there, in terms of their environmental impact.

The AASV 2.0 PRRSV Classification System (21) categorizes the PRRSV infection status of pig farms. This system is mainly based on PCR and serology. For sow farms (their Tables 1, 2), the *positive unstable* statuses IA and IB involve PCR-detectable viral RNA in more than 25% (IA: *high prevalence*) or less than 25% (IB: *low prevalence*) of PCR-tested weaning-age piglets. The *positive stable* status II requires a 90-days absence of viral RNA in weaning-age piglets. The statuses *provisional negative* (status III) and *negative* (status IV) require the absence of ELISA-detectable antibodies (which indicate previous exposure to the virus) in replacement gilts 2 months after introduction into the farm (III) and in adult sows (IV). For groups of WtF pigs [(21), their Table 3], the AASV statuses include *positive* (tested ELISA-positive for antibodies and PCR-positive for viremia), *seropositive non-shedding* (tested positive for antibodies and negative for viremia), *negative* (tested negative both for antibodies and for viremia), and *vaccinated*. A group of WtF pigs may move from one status to another one during the growing period, typically from *negative* to *positive*.

In our LCA, the following three conceptual PRRSV infection phases were assigned within each sow farm following (22). Unlike the dynamic AASV statuses, this compartmental approach is not based on PCR or serology; rather, it defines production-centered snapshots, focusing on the impact of PRRS on production performance traits at different times associated with an outbreak registered in the POMP data.

*Negative* phase, 21 weeks (i.e., one sow reproductive cycle) to 2 days before the outbreak; roughly corresponding to the AASV statuses II, III, or IV. These sow farms wean PCR-negative pigs. An alternative label for this phase would be *Healthy*.

*Positive epidemic* phase, the first 16 weeks after the outbreak, when reproductive performance is impacted most seriously; roughly corresponding to the AASV status IA. These sow farms have recently experienced an outbreak; infection rates and viral shedding remain persistently high in the piglet population. Alternative labels for this phase would be *Affected* or *Sick*.

*Positive endemic* phase, 17–38 weeks after the outbreak (21 weeks, like the *Negative* phase, to cover the impacts of the virus throughout the mating, gestation and lactation stages); roughly corresponding to the AASV status IB. At least 75% of weaned piglets from these sow farms are PCR-negative. An alternative label for this phase would be *Recovered*.

Following this, a conceptual PRRSV infection phase was assigned to each WtF closeout, based on the sow farm infection phase and on the WtF farm’s clinical PRRS outbreak reports, as follows.

*Negative—negative phase*: closeouts that are PCR-negative at entry and remain so.

*Negative—positive phase*: closeouts that are PCR-negative at entry and experience an outbreak later in the WtF stage so that they are PCR-positive when delivered at the farmgate.

*Positive—positive phase*: closeouts that are PCR-positive at entry and remain so.

**Figure 1:**
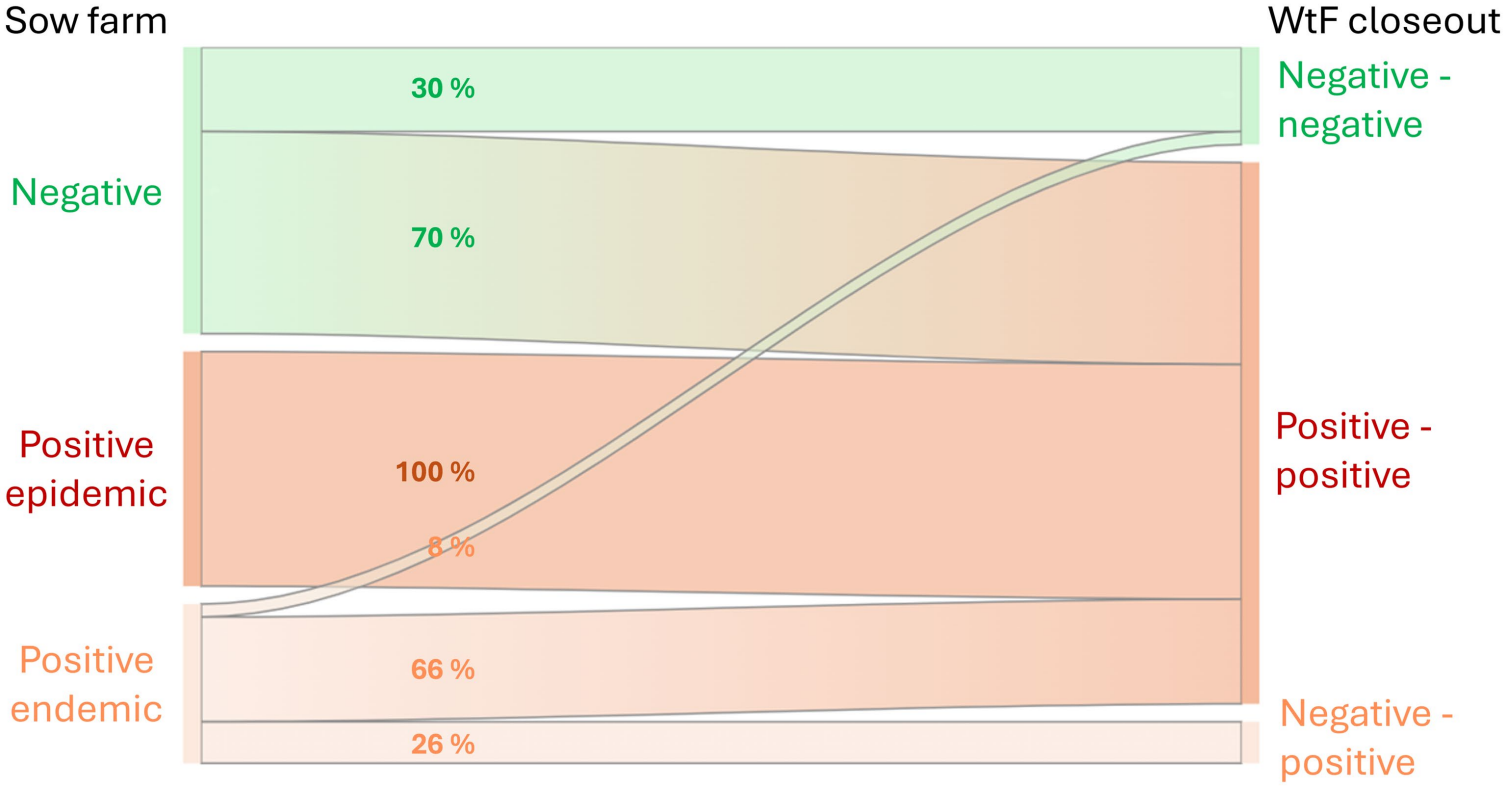
Pathways from sow farms in their three potential PRRSV infection phases to wean-to-finish (WtF) closeouts in their three potential infection phases. The WtF labels (… - …) stand for negative (or positive) at entry and negative (or positive) at delivery for slaughter. Note that the numbers on the links are proportions within each sow farm infection phase.

In the snapshot of the production system that an LCA must reflect, the population size of each of the infection phases is assumed to be stable over time. This leads to our conceptual framework of the effect of PRRSV on performance traits at the national steady-state scale, shown in Figure 1 with its six infection phase pathways that are modeled in our LCA. See section 2.4 for the pathway frequencies on the links in Figure 1.

The recorded data were standardized by the number of weeks in each phase, and then annualized to produce the input to the LCA analysis. Statistics for each trait were calculated per infection phase and compared within farm, i.e., each farm was compared to itself across the various phases.

### 2.3 Performance data by infection phase

Tables 1, 2 show the reproduction and production performance levels in the POMP data, by infection phase. At the sow level, compared to the *Negative* infection phase, the *Positive epidemic* phase is characterized by a 40% reduction of the number of piglets weaned per sow per year, through reductions of all its component traits. The *Positive endemic* phase is somewhere in between. At the WtF level, the most important effect of PRRSV infection is an increase of the mortality rate by 63% (*Positive—positive*) or 28% (*Negative—positive*); growth rate and feed efficiency are influenced too but less dramatically so.

**Table 1:** Reproduction performance traits (mean ± standard deviation) of 113 North American sow farms in the POMP data by infection phase, and frequencies (April 2024) of each infection phase among sow farms in the SHMP data.

Trait	Sow farm infection phase			Number of outbreaks
	Negative	Positive epidemic	Positive endemic	
TNB	14.1^a^ ± 0.87	13.8^b^ ± 0.94	14.0^ab^ ± 0.93	173
SB&M	1.36^a^ ± 0.38	2.78^b^ ± 1.06	1.61^c^ ± 0.41	158
PWM	0.150^a^ ± 0.10	0.236^b^ ± 0.12	0.169^a^ ± 0.11	172
SMRT	0.130^a^ ± 0.042	0.146^b^ ± 0.062	0.140^ab^ ± 0.042	125
Wean/wk	1787^a^ ± 964	1285^b^ ± 852	1587^a^ ± 852	173
PSY	25.9^a^ ± 4.4	15.3^b^ ± 5.4	25.2^a^ ± 4.1	172
Frequency	0.42	0.35	0.23	173

Means in a row that do not share a superscript letter differ significantly (*p* < 0.05) from each other. TNB, litter size (total number born); SB&M, number stillborn including mummies; PWM, preweaning mortality rate; SMRT, yearly sow mortality rate; Wean/wk, number of piglets weaned per farm per week; PSY, number of piglets weaned per sow per year.

**Table 2:** Production performance traits (mean ± standard deviation, number of closeouts in brackets) of North American wean-to-finish closeouts in the PROSPER data by infection phase.

Trait	Closeout infection phase (status at entry—at delivery for slaughter)		
	Negative—negative	Positive—positive	Negative—positive
AGE0	20.2^a^ ± 2.4 (3575)	21.1^b^ ± 2.8 (371)	20.0^c^ ± 2.4 (1634)
WT0	6.3^a^ ± 0.68 (480)	5.9^b^ ± 0.66 (108)	6.3^a^ ± 0.62 (1109)
WT1	129.5^a^ ± 8.2 (3568)	128.1^b^ ± 8.9 (386)	129.7^ac^ ± 9.0 (1618)
ADG	0.737^a^ ± 0.051 (3626)	0.716^b^ ± 0.057 (386)	0.724^c^ ± 0.049 (1638)
FCR	2.47^a^ ± 0.63 (542)	2.51^ab^ ± 0.48 (108)	2.56^b^ ± 0.48 (1147)
WFMRT	0.091^a^ ± 0.048 (3626)	0.149^b^ ± 0.093 (386)	0.116^c^ ± 0.059 (1638)
Frequency	0.18	0.75	0.08

Means in a row that do not share a superscript letter differ significantly (*p* < 0.05) from each other. AGE0: age at entry (days); WT0: weight at entry (kg); WT1: weight at exit (kg); ADG: average daily gain (kg/day); FCR: feed conversion ratio (kg feed/kg gain); WFMRT: wean-to-finish mortality rate; Frequency: estimated proportion of the nation-wide produced amount of live weight [from equation (1)].

### 2.4 Scaling the data

The objective of this study is to quantify its deliverables at the level of the entire North American pig production sector, and the functional unit of our LCA (see section 2.5) is defined in terms of liveweight. Hence the POMP data must be transformed to the nation-wide distribution of the infection phases, and the numbers of animals must be transformed to liveweight.

At the sow level, we used the distribution that is reported weekly for about half the USA sow population by the University of Minnesota’s Morrison Swine Health Monitoring Program (SHMP) (25).^3^ The SHMP values reported for April 2024, in terms of the infection phases used in our LCA, are in Table 1.

For the WtF level, we assume that pigs sourced from a sow farm in the *Positive epidemic* phase stay positive. The proportions of animals in the other pathways were quantified based on (7, 26–28), and on expert opinions of C. A. Corzo (University of Minnesota), J. Fent (Pig Improvement Company), D. C. P. Linhares (Iowa State University), and G. S. Silva (Iowa State University) (personal communications, 2024). The resulting pathway frequencies appear as the percentage values on the links in Figure 1.

Numbers of animals were transformed into liveweight by equation (1).


$${\mathrm{LWT}}_{\mathrm{ij}}=N\times p_{i}\times {\mathrm{PSY}}_{i}\times q_{\mathrm{ij}}\times (1–{\mathrm{MRT}}_{j})\times {\mathrm{LWT}}_{j}$$ (1)

Here, LWT_ij_ is the total liveweight (kg) of finisher pigs sourced from sow farms with infection phase i (*Negative, Positive epidemic,* or *Positive endemic*) and delivered at the farmgate after infection phase j (*Negative—negative*, *Negative—positive*, or *Positive—positive*); *N* = 6.23 million is the size of the USA sow population; p_i_ is the proportion of the sow population in infection phase i (from Table 1); PSY_i_ is the average number of piglets weaned per year per sow in infection phase i (from Table 1); q_ij_ is the proportion of pigs sourced from sow infection phase i that end up in WtF infection phase j (from Figure 1); MRT_j_ is the average WtF mortality rate among pigs in infection phase j (from Table 2); LWT_j_ is the average liveweight at delivery of finisher pigs in infection phase j (from Table 2).

The resulting LWT_ij_ values sum to the WtF_j_ infection phase frequencies in the bottom row of Table 2.

Individual sow farms usually progress from the *Negative* phase via *Positive epidemic* to the *Positive endemic* phase, and these phases typically last for less than a year each. The conceptual model underlying our LCA is based on the assertion that, although an individual farm moves through different infection phases over time, at the scale of the whole North American production sector there is an approximately stable number of farms within each phase. This allows for a representative description of the overall impact of PRRS even though the LCA does not include a dynamic model to simulate the time series behavior of individual farms. The six pathways of Figure 1 are each represented in the lifecycle inventory model as a WtF farm with performance levels as in Table 2, sourced from a sow farm with performance levels as in Table 1. The six sets of LCA results were then weighted into the North American industry average by the LWT_ij_ values from equation (1).

### 2.5 Life cycle assessment

The aim of our study is to quantify the effect of PRRSV elimination on several environmental impact categories, via Life Cycle Assessment (LCA). We compare North American farms in the *Negative* infection phase to the North American industry average which is a mix of *Negative*, *Positive epidemic* and *Positive endemic* farms as quantified at the end of section 2.4, and in another analysis we compare *Negative* farms to *Positive epidemic* and *Positive endemic* farms only (representative of the status of large parts of midwest USA, central Mexico, and mid-east China). The LCA methodology described in this section is identical to the methods followed by (29); it passed critical and independent peer review to demonstrate conformance with the recommendations and guidance within the ISO standards 14,040:2006, 14,044:2006, and 14,071:2014 (30, 31).

#### 2.5.1 Functional unit, system boundary, cutoff criteria, and multi-functionality

The functional unit of this study is the delivery of 1,000 kg pig live weight at the farmgate; this includes culled sows from the upstream breeding herd. It does not include any animals not fit for slaughter; these do not enter the food chain (no live weight delivered, no desirable output), but their resource consumption, their emissions, and other undesirable outputs are included in the calculations. We assumed identical animal husbandry practices, farm infrastructure, post-farm activities, and ancillary activities such as accounting and travel for the systems being compared. Our system boundary [(29); their Figure 1] begins with the extraction of raw materials and includes all animal husbandry activities, feed production (production of fertilizer, pest and disease control chemicals, seed, fuel, and energy), manure management, and veterinary and genetic research and development (R&D) activities. No simplifying cutoff criteria (31) are implemented.

The primary multi-functional processes are the parent, grandparent (GP), and great-grandparent (GGP) stages (collectively known as the genetic pyramid; the “parent” is the parent of the slaughter pig) from where culled sows (a coproduct) are sent to slaughter; all environmental impact from these stages is assigned to the progeny, and an internal credit for the displaced growing-finishing pigs is assigned to the GP and GGP sow barns (similar to a consequential LCA approach to avoid allocation). Likewise, male piglets produced in the GP stage form another coproduct; they are sent to finishing, where they displace piglets from the parent sow barn. Thus, the live weight at the farmgate accounts for all the animals leaving the system to slaughter [(29); their Figure 1].

An inventory of inputs and emissions is created for each activity and constructs the cradle-to-farmgate lifecycle inventory (LCI) model, linked from the GGP stage to the finishing stage.

#### 2.5.2 Unit processes and their data requirements

The foreground system includes the main activities that deliver the functional unit of Section 2.5.1, i.e., a typical North American commercial pig production operation from gestation and farrowing facilities to finishing and slaughter. To simulate this operation and estimate the utility consumption, we used the Pig Production Environmental Calculator of the University of Arkansas Resiliency Center (32). The primary data required are listed in Supplementary Table S1. We used the market group process for USA electricity (Ecoinvent V 3.9.1 cutoff system model) and the I/O model by Carnegie Mellon University (33) for veterinary and genetic R&D services. Environmental impacts associated with infrastructure such as buildings and machinery were not included for foreground processes except when an existing Ecoinvent (see the next paragraph) unit process includes infrastructure.

Emissions associated with manure management (ammonia, methane, non-methane volatile organic compounds, and nitrous oxide) are not commonly measured and were therefore modelled. This requires estimates of manure excretion and composition; we followed the LEAP recommendations (accessed on 09 November 2025)^4^ to treat manure as a residual, consistent with the treatment of similar products in the background. The most relevant data gap in this study is the industry-wide distribution of systems for manure management and subsequent application; we modelled a uniform use of deep pit systems, as used in a significant fraction of North American production systems.

The background system includes all upstream activities. Purchased inputs to the main production processes such as electricity, fuel, and transportation were sourced from the Ecoinvent v3.9.1 cutoff database (34) (accessed on 09 November 2025);^5^ some feed ingredients were sourced from the Agrifootprint 5.0 database (35, 36). In North America, activities downstream of the abattoir are not affected by genetic factors, so we excluded them from the system boundary.

#### 2.5.3 Diet composition

Diets for sows and for growing-finishing pigs were formulated according to the nutrition and feeding guidelines of PIC (37), based on ingredients typically used in North America. The amounts of sow feed consumed in the gestation and lactation phases were estimated with PIC’s Dynamic Sow Feeding Tool (accessed on 09 November 2025).^6^ For the industry average evaluation, the same diets and computational procedures were used, averaging the feeding guidelines of PIC and of other breeding companies in North America because the industry average data does not specify the genetic background of its pigs. Supplementary Table S2 gives the diet compositions. For growing-finishing pigs, diet composition was derived from the growth rate and feed conversion performance reported in Table 2. The allocation per phase was determined by the weaning body weight, market body weight, and overall feed conversion using the PIC Feed Budgeting tool (accessed on 09 November 2025^7^; overall growth rate 0.77 kg/d, overall FCR 2.55 kg/kg). Similar procedures were used for the industry average to adjust for the differences in growth rate and feed conversion (0.75 kg/d, 2.71 kg/kg). Supplementary Table S3 gives the diet compositions.

Differences in mortality affected FCR because dead pigs were attributed feed consumption but no market weight output (see section 2.5.1). There is little published evidence of impacts of infectious diseases such as PRRS on FCR beyond the direct effect of mortality (38) (p. 231). The same holds for impacts of immune system activation on the requirements for amino acids and other nutrients beyond the impacts on feed intake as such (39–41). Our modeling accounted for the differences in feed intake of PRRSV-infected pigs and adjusted the dietary phase feed budgets; but nutrient contents were kept constant across the diets within each phase.

#### 2.5.4 Lifecycle impact assessment

LCIA frameworks consist of a series of category-specific characterization factors used to convert the cumulative inventory results of an LCI model to the cumulative impact in multiple environmentally relevant categories for the functional unit of the system under study. For example, the climate change impact is calculated by taking the product of each greenhouse gas emission and its respective characterization factor to convert the cumulative, relative radiative forcing into carbon dioxide equivalents (CO_2_eq). We use three LCIA frameworks to characterize the environmental impact of the functional unit—not to expressly investigate the differences between them (that is outside the scope of this study), but to quantify the sensitivity of our results to their various underlying assumptions and points of view which is a requirement of the ISO guidelines (31). These frameworks are as follows.

*ReCiPe-2016* includes 18 impact categories covering the areas of protection identified under human health, ecosystem health, and resource conservation. It includes a 2010 world-based normalization method that can aid in defining which categories are relatively larger in the context of the annual average global per capita emissions. We apply all three of the cultural perspectives of ReCiPe-2016: Egalitarian, Hierarchist, and Individualist.

*Environmental Footprint-3.1* is the framework required for the Product Environmental Footprint (PEF) recommended by the European Commission.

*IPCC-2021* climate change includes 12 climate-related impact categories. Its category of primary interest for this study is the 100-year global warming potential (“GWP-100”), as it coincides with the global warming categories in ReCiPe and PEF. Other categories include global warming potential for different time horizons and global temperature change potential.

The lifecycle impact assessment (LCIA) calculations were performed using openLCA v2.02 to link the individual stages of production, creating a supply chain model to convert the material and energy flows of pig production to the impact categories of the three frameworks. Background data were separately licensed from the Ecoinvent v3.9.1 cutoff and APOS system models and the Agri-footprint 5.0 databases; this includes the probability distributions for emissions of ammonia and greenhouse gases from crop production. We adopted the uncertainty distributions of the background database without revision.

#### 2.5.5 Contribution analysis

The contributions of the various processes to each impact category were quantified for the comparison of the *Negative* infection phase to the industry average. The inventory projection focuses only on the production traits of the production systems; it does not include projected changes in efficiency or other contributing sectors such as electricity, crop yield, or transportation. Hence we have not performed a separate contribution analysis for the comparison of the *Negative* infection phase to the *Positive epidemic* and *Positive endemic* phases only.

#### 2.5.6 Methodological quality assessment

LCA has several sources of uncertainty that can influence the interpretation of the results and limit the conclusions (42, 43). These include (i) natural variability in input parameters such as fertilizer or fuel use, (ii) estimated values obtained through proxy sources, substituting a similar product for one that does not exist in available databases, and (iii) results from mathematical models that include multi-year simulations of parameters associated with variable factors such as weather or soil conditions. There are also uncertainties in impact assessment characterization factors, but the studied systems are quite similar; therefore, we expect these to be uniform across all compared systems and not affect the study conclusions. Nonetheless, the selection of an LCIA framework is a value choice and should be tested for sensitivity. Comparison of the results from the ReCiPe and the PEF frameworks (section 2.5.4) evaluates the robustness of the results.

We quantified the uncertainty in the LCA results using Monte Carlo simulation (MCS), available in the openLCA software. This process is rules-based and incorporates prior probability distributions for uncertain or variable input data that reflect the knowledge and process uncertainty associated with the variables and describe the range of expected or permissible parameter values. We applied log-Normal prior probability distributions to avoid sampling of negative input values: most of the reference flows in LCA are positive definite, so negative values are not permissible. Confidence limits for log-Normal probability distribution functions follow from Burnham et al. (44) (p. 212) and Buckland et al. (45) (pp. 88–89) as *μ* × C (lower limit) and μ/C (upper limit), where C = $\exp[z_{\alpha }\sqrt{\ln(1+\sigma^{2}/\mu^{2})}]$ , μ and σ^2^ are the mean and variance of the reported Normal statistics, z_α_ is the standardized Normal variate for the desired confidence level (e.g., 1.96 for 95% confidence), exp.(⋅) is the exponential function, and ln(⋅) is the natural logarithm.

MCS produces posterior probability distributions for its predicted output variables (i.e., the impact categories), which can be used for statistical significance testing. We used the GLM procedure of SAS 9.4 (46) to perform a two-sided unpaired ANOVA on sets of 100 MCS results for each impact category to test the significance of the contrasts among the PRRS-negative, endemic and epidemic production systems. We used SAS procedure UNIVARIATE to test for normality of the data distribution.

Characterization factors are used to translate the inventory to the impact categories. The magnitude of an observed contrast is not necessarily indicative of its statistical significance: a factor of two for climate change may be significant, while a factor of 1,000 for toxicity effects may not, partially due to the much larger range in characterization factors for toxicity. An additional explanatory factor arises in the process of uncertainty propagation in the MCS when the uncertainty of some of the contributing flows is higher, resulting in a wider frequency distribution in the MCS and therefore less differentiating power in the statistical tests. The flow uncertainty for foreground processes was quantified via the Ecoinvent pedigree matrix: five characteristics of the data (reliability, i.e., how was it measured; completeness; temporal correlation, i.e., how recent is it; geographical correlation, i.e., where is it from; technological correlation, i.e., which technology generated it) are qualitatively assessed in terms of five indicator scores, scored from 1 for the highest quality, e.g., “verified data based on measurements,” to 5 for the lowest quality, e.g., “non-qualified estimate” [(47); their Table 10.4]. These are converted into five default variance values [(47); their Table 10.5], which increase from zero to higher values for more unfavorable indicator scores. Each of these was combined with its corresponding basic uncertainty as estimated from aggregate statistics for the production traits. This defines the prior variance of the contributing flow as it goes into the MCS. In the inventory analyzed here, all elements had indicator scores of [1 1 1 1 1].

Our uncertainty assessment did not include uncertainty associated with the characterization factors. Because the systems being compared are very similar, this omission of a source of variability in the results will not introduce bias in the conclusions; any single MCS would have selected the same characterization factor for both systems.

## 3 Results

Fourteen of the impact categories covered by the ReCiPe-2016 and PEF-3.1 frameworks have functional overlap across the frameworks, in the sense that they are qualitatively the same but may have different quantitative reporting units: global warming, acidification, the use of fossil fuel resources and mineral resources, freshwater ecotoxicity and eutrophication, marine eutrophication, fine particulate matter formation, ozone depletion, water and land use, carcinogenic and non-carcinogenic toxicity, and ionizing radiation. The GWP-100 category of IPCC-2021 coincides with those global warming categories. Of these impact categories, global warming and water use are of significant societal concern. Fossil fuel use, land use, fine particulate matter formation, and freshwater eutrophication are categories with reasonably available and reported high-quality data. Acidification is generally much less of a concern today than it was in the past (in USA and Europe because of significant reductions in acidifying emissions due to the Clean Air Act and the Ambient Air Quality Directive and its successors, respectively); the impacts of human toxicity and ecotoxicity and of marine eutrophication are dominated by factors that are not significantly influenced by the alternative scenarios evaluated here. Therefore we focus here on the six impact categories listed in Table 3.

**Table 3:** Focal environmental impact categories, as defined by the ReCiPe-2016 and PEF-3 frameworks.

Impact category	ReCiPe-2016	PEF-3
Global warming	Global warming potential of GHG emissions on a 100-year time horizon, in kg CO_2_-equivalents	Radiative forcing as global warming potential (GWP100), in kg CO_2_-equivalents
Fossil fuel resources	Depletion of fossil fuel resources, in kg oil-equivalents	Non-renewable abiotic depletion potential, net calorific value in MJ
Freshwater eutrophication	The increase in phosphorus mass per kg P discharged to aquatic environments, in kg P-equivalents	Fraction of nutrients reaching freshwater compartment (P), in kg P-equivalents
Fine particulate matter formation	Disease incidence due to 1 kg of emitted particulates smaller than 2.5 μm, in kg PM_2.5_-equivalents	Impact of particulate matter on human health: disease incidence
Water use	Water consumed by a unit process and thus no longer available in a watershed, in m^3^	User deprivation potential, deprivation-weighted water consumption, in m^3^
Land use	Assess how land use and its change affect biodiversity, in m^2^ × year annual crop equivalents	LU/LUC radiative forcing as Global Warming Potential (GWP100), in kg CO_2_-equivalents

The impact estimates are presented not as absolute values but proportionally: we report the results for *Negative* farms as proportions of the industry average results, or of *Positive* farms. Many of the overlapping ReCiPe-2016 and PEF-3 frameworks have different quantitative reporting units across the frameworks, and this proportional approach makes them quantitatively comparable. For example, fossil fuel resources are quantified in terms of oil equivalents (in kg) by ReCiPe-2016 but in terms of net calorific value (in MJ) by PEF-3; this is resolved when both are transformed to a proportion of a similar associated reference value. Another important advantage of the proportional approach is that it is considerably less sensitive to external factors such as feeding levels, feed composition and manure management systems, and to the underlying assumptions and points of view of the LCIA frameworks.

In all these cases, a lower environmental impact is more favorable, but to stay conservative we applied two-sided significance tests. Shapiro–Wilk tests confirmed normality of the data distribution in all cases (*p* > 0.31). Levene tests for unequal group variances were significant (*p* < 0.05) in 56% of the cases, and we report Welch variance-weighted *p* values there; in the most extreme case, this changed the *p*-value from 0.00100 to 0.00107.

### 3.1 PPRSV-negative systems versus industry average

**Figure 2:**
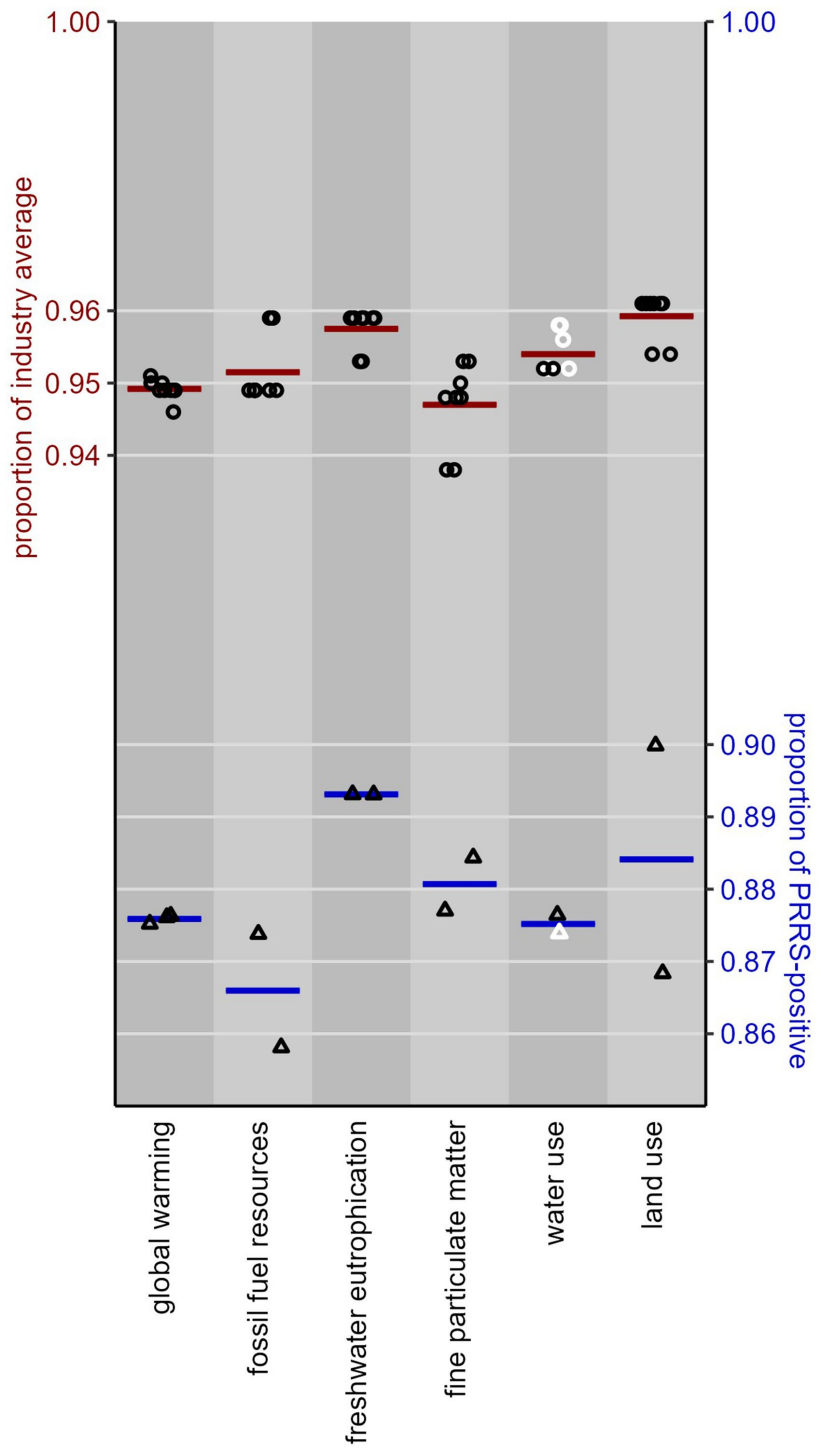
Upper plot (circles, red lines): performance of North American pig production systems in the *Negative* PRRSV infection phase as a proportion of the performance of the North American industry average, for six focal environmental impact categories, as modeled in 10 LCIA frameworks (ReCiPe-2016 Egalitarian, Hierarchist and Individualist, cutoff and APOS; PEF-3.1 cutoff and APOS; IPCC-2021 cutoff and APOS). Lower plot (triangles, blue lines): ditto for the proportion of the performance of PRRSV-positive systems, as modeled in ReCiPe-2016 Hierarchist cutoff, PEF-3.1 cutoff, and IPCC-2021 cutoff. Each datapoint represents an LCIA framework; each horizontal line gives the mean of its associated datapoints. Lower values are more favorable; black (or white) datapoints indicate if the estimated proportion is (or is not) significantly (*p* < 0.05) different from 1 (i.e., from the industry average, or from PRRSV-positive systems).

Our comparison of the performance of PPRSV-negative pig production systems to the North American industry average [pathway *Negative* to *Negative—negative* of Figure 1 versus all its pathways, weighted by the LWT_ij_ values from equation (1)] shows a significantly (*p* < 0.05) lower environmental impact for 14 of the 18 impact categories in the ReCiPe-2016 framework, for 18 of the 25 categories in the PEF-3.1 framework, and all 12 climate change categories in the IPCC-2001 framework. All these results are presented in Supplementary Data Sheet 2. The *Negative* system has 4–6% lower environmental impact than the North American industry average for the 14 categories common to ReCiPe-2016 and PEF-3. The upper plot in Figure 2 shows the results for our six focal impact categories. Water use is the only of these categories where some of the LCIA frameworks produced non-significant differences between *Negative* farms and the industry average, but the estimates as such are indistinguishable from the other ones.

The 10 LCIA frameworks produced very uniform results, especially for global warming, acidification and ozone depletion; for most of the other categories the PEF-3.1 framework scores 0.6–1.2 percentage points higher (the use of fossil fuel resources and mineral resources, and water use) or lower (ecotoxicity, freshwater and marine eutrophication, and land use) than ReCiPe-2016. The only significant differences among the frameworks are for the use of fossil fuel resources, ecotoxicity and marine eutrophication (*p* < 0.014).

### 3.2 PRRSV-negative versus PRRSV-positive systems

Our comparison of the performance of PRRSV-negative pig production systems to PRRSV-positive ones (pathways *Negative* to *Negative–negative* of Figure 1 versus its three pathways *Positive epidemic* to *Positive–positive*, *Positive endemic* to *Positive–positive*, and *Positive endemic* to *Negative–positive*) shows a significantly (*p* < 0.05) lower environmental impact for 17 of the 18 impact categories in the ReCiPe-2016 framework, for 17 of the 25 categories in the PEF-3.1 framework, and all 12 climate change categories in the IPCC-2001 framework. All these results are presented in Supplementary Data Sheet 2. The *Negative* system has 9–17% lower environmental impact for the 14 categories common to ReCiPe-2016 and PEF-3.1. The lower plot in Figure 2 shows the results for our six focal categories. Again, water use is the only of these categories where one of the LCIA frameworks (PEF-3.1) produced a non-significant difference, but the estimate as such is indistinguishable from the other one. These mean impact estimates in Figure 2 (blue lines) vary much more across the impact categories than those of section 3.1 (red lines), due to a buffering effect of the *Negative* pathways included in the industry average.

The results differ very little between LCIA frameworks, in a similar way as in section 3.1. The largest difference (10% vs. 13%) is for land use, but this difference is not statistically significant (*p* = 0.38). Smaller differences for marine eutrophication (10% vs. 11.5%) and ozone depletion (10.8% vs. 11.4%) were statistically significant (*p* < 0.001); for all other impact categories, the frameworks produced essentially identical results (0.08 < *p* < 0.9).

### 3.3 Contribution analysis

For ReCiPe-2016, the animal-rearing phases (finisher and piglet) are major contributors only to terrestrial acidification and fine particulate matter formation; both are dominated by ammonia emissions. Corn production is the dominant contributor across many impact categories; its primary contributing factor is emissions associated with fertilizer application. Metals, extracted and consumed in the supply chain, and fossil fuel consumption are the obvious dominant contributors to mineral resource and fossil fuel resource scarcity, respectively. The ionizing radiation category is dominated by waste management, with formaldehyde production as the primary contributing factor.

PEF-3.1 largely repeats the ReCiPe-2016 patterns, with interesting exceptions in the categories ozone depletion, land use, water use (where corn production is the dominant contributor in ReCiPe but is very minor in PEF), and in carcinogenic toxicity (where the opposite holds). In ReCiPe, land use is an indicator of potential impact on biodiversity associated with different land use classifications; in PEF the main contribution is dominated by infrastructure such as buildings and roads, and to a lesser extent forestry. The primary “waste management” contributing factor to ionizing radiation in PEF-3.1 is polystyrene.

## 4 Discussion

In this study we quantified the influence of PRRSV infection on the environmental impact of the North American pig production sector. This influence is mediated by the detrimental effect of PRRS on mortality and (re)production traits in sows and WtF pigs; this worsens a farm’s whole-enterprise feed conversion ratio, which is strongly correlated to nitrogen excretion—the main contributor to the GHG emission from monogastric mammals and hence to their contribution to global warming. Similar associations hold for other environmental impacts. Our LCA results show that, in North America, the environmental impact of PRRSV-negative production systems is 4–6% lower than the impact of the industry average, and 9–17% lower than the impact of PRRSV-positive systems.

### 4.1 Methodology

Our environmental impact estimates are influenced by several aspects of the methodology applied, either positively (overestimation) or negatively (underestimation).

As mentioned in section 2.1, a criterion for a farm’s eligibility for inclusion in the POMP project that provided our data is that it plans to eliminate PRRSV from the weaning population in case of stable vaccinated farms, or from the whole herd in case of naive farms. Farms without such plans decide to coexist with the virus and aim to return to baseline production; their production performance levels are typically more similar across the *Negative, Positive epidemic,* and *Positive endemic* infection phases of our study, in the sense that performance in the *Negative* phase tends to be less favorable than what is listed in our Table 1. Inclusion of such farms in the data analyzed in studies like ours would then reduce the contrast between the infection phases and hence reduce the estimated environmental impact of PRRSV elimination (a case of overestimation).

As mentioned in section 2.1, PRRS outbreaks with concurrent PED outbreaks had been removed from our data; this typically involves about 5% of PRRS outbreaks. PED can cause extremely high preweaning mortality rates, and USA farms experiencing PED commonly depopulate either the farrowing rooms or the whole farm; this changes the sow population significantly and disrupts performance data recording, so it makes the farm useless for the purposes of our study. PRRS and PED both influence preweaning mortality; concurrent outbreaks would cause confounding and overestimation of the environmental impact of PRRS. This makes our impact estimates conservative (underestimation).

PRRSV-positive farms are frequently reinfected with another variant of the virus for which the population has no immunity (48). This will cause the farm to take longer to return to its baseline production levels and/or to suffer more intense production losses (49)—and this will increase its environmental impact. The POMP data did not allow for estimating the effects of such rebreaks and this makes our impact estimates conservative (underestimation).

PRRSV infection reduces the digestibility of dry matter, energy and protein from the feed (50) and it increases the nitrogen content of manure (16); this will increase nutrient excretion and therefore environmental impact. Our models could not take this into account, which makes our results conservative (underestimation).

As mentioned in section 2.5.2, our analyses modeled a single manure management system (the deep pit system used in a significant fraction of North American pig production). Alternative management systems would lead to different absolute values for the estimated impacts; this is another reason for reporting our results as proportions from a reference value. In another LCA study [a follow-up of (29), now submitted for publication and in revision] we did model two manure management systems: (i) the typical Chinese combination of composting, lagoons and anaerobic digestion, with an IPCC (51) methane conversion factor MCF = 0.1 and an IPCC (51) N_2_O emission factor EF3 = 0.01, and (ii) high efficiency low leakage anaerobic digestion with MCF = 0.01 and EF3 = 0.0006. In 159 of 182 cases, the proportional impact estimates for management system (i) were between 99 and 101% of the associated estimates for system (ii); the most extreme cases ranked as 96.6 and 103.6%. We conclude that our proportional impact estimates are insensitive to the manure management system modeled.

As mentioned in section 2.4 we asserted that at the scale of the whole North American production sector there is an approximately stable number of farms within each of the *Negative*, *Positive epidemic*, and *Positive endemic* infection phases, although an individual farm moves through the phases over time. The software used for our LCA does not include a dynamic model of the time series behavior of individual farms. Such a model might simulate the LCI inventory data for a farm in, for example, weekly time steps and run an LCA on that inventory for each week. An example is the Daycent-CR package described by Mathers et al. (52). This approach would provide an interesting (but logistically and computationally very demanding) follow-up of our current study. It could predict, for example, to what extent production performance is notably different in summer than in winter; this would enable accounting for *intra*-annual temporal variability which we had to ignore. Assuming that a sick animal under cold or heat stress will perform worse than a healthy animal under cold or heat stress, our proportional estimates are then again conservative. Temporal variability is a relevant driver of uncertainty on an *inter*-annual basis, but not in our LCA snapshot approach described in section 2.2. It could be modelled outside a dynamic modelling environment by varying the values of parameters p and q in equation (1); the difference between the outcomes of the scenarios modelled in sections 3.1 and 3.2 is an extreme example.

### 4.2 Interpretation

Our results seem conservative in comparison to the scarce literature on this topic. Capper (17) (her Table 10) modeled pig production systems with 60 and 10% PRRS prevalence and found GHG emission intensities at 8.19 and 6.35 kg CO_2_eq per kg carcass weight (i.e., a 22% reduction). Gombos et al. (53) studied five farms before and after the PRRSV elimination project in Hungary (see section 4.3) and reported a 1.95 kg/kg reduction of GHG emission intensity from their assumed baseline level of 6.1 kg CO_2_eq per kg carcass weight (i.e., a 32% reduction). Those authors used parameter values compiled from the literature and from public and local databases; many of these differ considerably from our values in Tables 1, 2, which were derived from an internally consistent North American dataset. Hence, even though different studies disagree quantitatively, PRRSV elimination clearly results in significant mitigation of environmental impact.

Gickel et al. (54) reviewed the literature on the effect of vaccination of growing-finishing pigs against three other important pathogens (porcine circovirus type 2 and the bacteria *Mesomycoplasma hyopneumoniae* and *Lawsonia intracellularis*) on their growth rate, feed conversion ratio and postweaning mortality rate. This produced data from 55 experiments comparing epidemically infected non-vaccinated control groups to epidemically infected vaccinated trial groups (equivalent to our comparisons of PRRSV-*Negative* farms to the industry average and to PRRSV-*Positive* farms). These production performance data were then used as input for an LCA with the Opteinics™ package (55) (p. 59) to estimate the carbon footprint in CO_2_eq per kg body weight gain. Their conclusion was that vaccination against these diseases can reduce the carbon footprint of growing-finishing pigs by 2.5–12.1%.

Similar studies on cattle and sheep were reviewed by Kyriazakis et al. (15); most of these focused on macroparasites and multifactorial health issues, but Skuce et al. (56) also included analyses of the effect on GHG emission in Scotland of the viruses that cause rhinotracheitis in cattle or adenocarcinoma in sheep, of the bacteria that cause Johne’s disease in cattle and sheep, abortion or footrot in sheep, or leptospirosis in cattle, and of the protozoa that cause neosporosis in cattle. They concluded that “some diseases will have more GHG abatement potential than others based on their prevalence, impact on infected animals etc. However, decisions on which diseases to prioritise for control and/or eradication must also take into account the cost-effectiveness and feasibility of GHG mitigation measures in practice.” From that same review (15), bacterial mastitis in dairy cattle also affects GHG emission. The quantitative disagreement between different studies mentioned in the first paragraph of this section was also signaled by (15) who concluded that “in general, modelling studies are not quantitatively consistent in their predicted impacts of a given health challenge on GHG emissions, and this reflects the differences in the assumptions and methodological choices upon which these models are made.”

The North American pig production sector emitted 70 Mton (i.e., 70 × 10^9^ kg) CO_2_eq in 2015 (GLEAM3, accessed on 09 November 2025).^8^ Based on the USA time trends of enteric CH_4_ and manure-management-related CH_4_ and N_2_O emissions reported by EPA [(57); their Tables 5.3 and 5.7] and EPA [(58); their Tables 5.3 and 5.6], this value can be extrapolated to 85 Mton for 2024. Our LCA results show that complete elimination of PRRSV in North America would then save 0.05 × 85 = 4.25 Mton CO_2_eq emission per year. This is half the total GHG emission of Luxembourg or of the USA state Rhode Island. From another point of view, it is equivalent to removing (the GHG emission of) a random 5% of the North American pig population, i.e., of 4.5 million pigs: close to the whole 2023 pig herd of the United Kingdom.

### 4.3 PRRSV elimination strategies

Conventional strategies to prevent PRRS or eliminate PRRSV include intensified biosecurity measures (ranging from the use of quarantine facilities to air filtration), gilt acclimation with intentional virus inoculation, herd closure and rollover (HCR), herd depopulation and repopulation (HDR), and vaccination. All these strategies carry considerable costs, often also for non-affected farms.

The cost of HDR was estimated at 460 EUR per sow in Ireland (59), coinciding with a repayment time of 2.1 years; even longer repayment times were reported for USA (26) and Germany (60). Nevertheless, successful regional PRRSV elimination attempts have been reported from Chile, Sweden, Minnesota and Hungary (61–64). Chile became PRRSV-positive again in 2013 and used HCR to become PRRSV-negative in 2023.

Thomann et al. (65) modeled PRRSV vaccination strategies on the farm level and found that a 1% increase in vaccine effectiveness (which, by their definition, reduces disease effects by 1%) increased farm profitability in Germany by 3 EUR per sow. The farm-level economics of PRRS vaccination in USA were analyzed by Linhares et al. (66), who found that preventive vaccination of sow farms can be profitable when the infection frequency is at least once every 2.1 years. The global PRRS vaccine market was valued at 1.2 billion USD in 2022, from where it has been predicted to double by 2028 (67). However, Hu et al. (68) noted that the effectiveness of PRRS vaccines is controversial and may result in inconsistent protection and inconsistent benefits for pig producers. The virus shows continuous changes in pathogenesis and antigenic profiles; in endemic areas its dominant field strains show periodic alterations via antigen drift, and farms are highly vulnerable to newly emerged viral genotypes. For example, Clilverd et al. (69) described a farm in Spain with routine PRRSV vaccination where fluctuations of the PRRS prevalence could be linked to two simultaneous evolutionary events of the virus: one strain of the virus mutated 25 amino acid residues in proteins that bind it to the pig’s tissue and in proteins that help it circumvent the pig’s antibodies, and a second strain emerged with much lower sensitivity to the pig’s antibodies against the first strain. On another farm, Clilverd et al. (70) observed a batch of sows treated with vaccine that was damaged by accidental exposure to high ambient temperatures (>35 °C); this resulted in a strong increase of the infection rate and mortality in their progeny and it also allowed for a 42% increase in the genetic diversity of the virus.

Genetic resilience of farm animals to infectious disease has long been envisioned as a convenient way to control or neutralize the problem (71, 72); present overviews of the field, and Bishop and Woolliams (73) show that breeding for disease resilience is not an easy task, mainly because it requires massive data recording in commercial (and infectious) conditions.

An early (2009) study of the resilience of pigs to PRRS (74) found much higher heritabilities for the numbers of piglets born dead or mummified in PRRS-epidemic phases than in PRRS-negative phases, with effectively zero genetic correlations between the two phases. Their genome-wide association study (GWAS) revealed six single nucleotide polymorphism (SNP) markers associated with the numbers of piglets born alive, dead or mummified, explaining 2–5% of the genetic variance of these traits in PRRS-epidemic phases; these associations were absent in PRRS-negative phases. Eight years later, Dekkers et al. (75) reviewed the further developed state of the art at that time and stated that the “host response of nursery pigs to PRRSV infection was found to have a sizeable genetic component under controlled experimental challenge studies. In particular, […] a putative causative mutation was identified in the GBP5 gene which is involved in innate immune response. […] The WUR SNP in this region can be used to select for pigs that are more resistant and resilient to PRRS.” This SNP (registered as WUR10000125) is a genomic marker described by (76, 77); they found that the homozygous favorable genotype reduced PRRS viremia (up to 21 days of age) by 4–5% and that it increased PRRS-challenged growth rate (up to 42 days of age) by 2–12%, compared to the heterozygote and the unfavorable homozygote combined. Khatun et al. (78) evaluated this SNP and two other ones associated with the GBP5 gene; they found similar results for viremia and growth rate as above, and also significant (*p* < 0.05) effects on (i) one of two tested cytokines and on (ii) 10 of 25 tested combinations of five types of T-lymphocyte white blood cell in five types of tissue. Chase-Topping et al. (79) carried out a controlled transmission experiment and could not find “evidence that genetic selection on the WUR genotype would affect PRRSV-2 transmission.”

This involved North American PRRSV Type-2 strains. Abella et al. (80) evaluated the WUR SNP against a European PRRSV Type-1 strain and found that “pigs carrying the G allele grow faster than those which are homozygous for the allele A after vaccinating them with an attenuated European PRRSV strain. The most striking result is that this effect is reversed in a PRRSV-free environment.” The latter finding is worrying because it implies that selection for this allele would have opposite effects on production traits in different PRRS infection phases, and this would make a commercial selection program very complicated. Dunkelberger (81) (her Table 7.4) could not replicate Abella’s finding in other (North American) pig populations.

The effects of another SNP marker (MARC0034894) on six sow reproduction traits of two breeds in the pre-PRRS, PRRS, and post-PRRS phases were analyzed by Hickmann et al. (82); out of the 6 × 2 × 3 = 36 effects estimated, five were significant (*p* < 0.05) and these estimates were contradictory across infection phases and breeds.

Dekkers et al. (75) further noticed that while such studies are “unlikely to identify pigs that are completely resistant to PRRS, they are starting to unravel the genetic basis of host response to PRRS, leading to the ability to select pigs that are less susceptible to PRRSV infection […], an additional and complementary approach to fight the impact of PRRS.” As a first step into that direction, in 2018 breeding company Topigs-Norsvin implemented the WUR SNP into its routine genomic breeding value estimation system,^9^ up to now (late 2025) without unequivocal published information about the effects in commercial conditions.

In summary, it is now clear that resilience of the pig to PRRSV has a firm genetic basis but that single genes with very strong effects do not play a dominating role in it; as with all traits with a complex biological background and very challenging data recording, the “black box” technology of classical selection (with or without genomics) is not likely to offer fast and complete solutions to the problem. You et al. (83) came to that same conclusion from their literature review. An alternative approach would be genome editing to produce animal populations fully resistant to a particular pathogen [e.g., (84)]. Petersen et al. (85) described how this can be exploited for sector-wide elimination of a disease. As the swine industry continues to innovate solutions to the PRRS virus, the results of the current study are relevant to any management, veterinary, genetic, or other innovation that can eliminate the PRRS challenge.

## 5 Conclusion

In North America, the environmental impact of PRRSV-negative pig production systems is 4–6% lower than the impact of the industry average, and 9–17% lower than the impact of PRRSV-positive systems. This forms an additional reason to eliminate the disease, over and above its effects on animal health and welfare, farm profitability, farm staff wellbeing, and public perception of pig production.

## Author’s note

This article is based on Thoma, G.J. (Environmental sustainability evaluation of PRRS-resistant production vs. industry average: North America; Resilience Services PLLC: Denver, CO, United States, 2024; https://tinyurl.com/5bn3kw3y, accessed on 09 November 2025), a life cycle assessment study that passed critical and independent peer review to demonstrate conformance with the recommendations and guidance within the ISO standards 14040:2006, 14044:2006, and 14071:2014.

## Data Availability

Publicly available datasets were analyzed in this study. This data can be found at: https://fieldepi.org/pomp, https://fieldepi.org/prosper.

## Footnotes

1
https://fieldepi.org/pomp

2
https://fieldepi.org/prosper

3
https://mshmp.umn.edu/reports#Charts

4
https://www.fao.org/partnerships/leap/en

5
https://ecoinvent.org/the-ecoinvent-association

6
http://dynamicfemalefeeding.pic.com/en

7
https://www.pic.com/resources/nutrition-links-and-tools

8
https://foodandagricultureorganization.shinyapps.io/GLEAMV3_Public

9
https://tinyurl.com/ajdn9tb2

## Glossary

**List of Acronyms**

AASV: American Association of Swine Veterinarians
ANOVA: Analysis of variance
CEM: Compartmental epidemiological model
CH_4_: Methane
CO_2_: Carbon dioxide
CO_2_eq: Carbon dioxide equivalents
EF3: N_2_O emission factor
ELISA: Enzyme-linked immunosorbent assay
EPA: USA Environmental Protection Agency
EUR: Euro (the currency)
FCR: Feed conversion ratio
GGP: Great-grandparent
GHG: Greenhouse gas
GP: Grandparent
GWAS: Genome-wide association study
GWP-100: 100-year global warming potential
H_2_S: Hydrogen sulfide
HCR: Herd closure and rollover
HDR: Herd depopulation and repopulation
IPCC: Intergovernmental Panel on Climate Change
ISO: International Organization for Standardization
LCA: Lifecycle assessment
LCI: Lifecycle inventory
LCIA: Lifecycle impact assessment
LEAP: Livestock Environmental Assessment and Performance
LWT: Liveweight
MCF: Methane conversion factor
MCS: Monte Carlo simulation
MJ: Megajoule
MRT: WtF mortality rate
N_2_O: Nitrous oxide
NH_3_: Ammonia
PCR: Polymerase chain reaction
PED: Porcine epidemic diarrhea
PEF: Product Environmental Footprint
PIC: Pig Improvement Company
POMP: PRRS Outbreak Management Program
PROSPER: Predictors of Swine Performance
PRRS: Porcine reproductive and respiratory syndrome
PRRSV: The virus that causes PRRS
PSY: Piglets weaned per sow per year
R&D: Research and development
RNA: Ribonucleic acid
SHMP: Morrison Swine Health Monitoring Program
SNP: Single nucleotide polymorphism
USD: United States dollar
WtF: Wean-to-finish production phase
