# Supplementary material for: The effects of PRRS on the environmental impact of pig production: a life cycle assessment study
Source: Front Vet Sci. 2025 Dec 11;12:1625581. doi: 10.3389/fvets.2025.1625581 (PMC12739953; doi:10.3389/fvets.2025.1625581)
Supplement: Supplementary file 1 [file Data_Sheet_1.pdf]

Table S1. Diet compositions (%) for the sow farm.

	gestation	lactation
Corn	59.156	44.928
DDGS	30.000	30.000
Soybean meal	7.034	17.439
Soybean oil	-	4.004
Limestone 36%	1.775	1.677
Monocal 21.0%	0.912	0.731
Salt	0.500	0.316
Vitamin-mineral premix	0.225	0.225
L-Lysine HCl 78.8%	0.230	0.440
Choline chloride 60%	0.127	0.127
Threonine 98.5%	0.041	0.094
Tryptophan 98.5%	-	0.012
Methionine 99% DL	-	0.007
Feed intake (kg/sow/year)	863	338

Table S2. Diet compositions (%) for the post-weaning nursery (N1 to N3) and finisher (F1 to F5) phases.

Ingredient	Post-weaning phase[1]							
	N1	N2	N3	F1	F2	F3	F4	F5
Corn	39.67	43.3	58.64	57.63	55.33	63.45	74.78	82.16
Oat Groats	12.5	10	-	-	-	-	-	-
Corn DDGS 6-9% oil	-	-	5	15	20	20.4	10.02	3.7
Extruded soybean meal	8.23	6.18	-	-	-	-	-	-
Soybean meal 47%	17	20	29.12	24.66	17.03	13.79	12.98	12.03
Fish meal	5	5	-	-	-	-	-	-
L-Lysine HCl 78.8%	0.54	0.49	0.54	0.38	0.40	0.35	0.28	0.25
Methionine 99% DL	0.28	0.26	0.23	0.09	0.02	-	-	-
Threonine 98.5%	0.25	0.23	0.24	0.09	0.06	0.04	0.04	0.04
Tryptophan 98.5%	0.06	0.06	0.06	0.01	0.02	0.02	0.01	0.01
Valine 96.5% L	0.10	0.09	0.12	-	-	-	-	-
Whey dried food grade 72%	13.72	10.98	-	-	-	-	-	-
Soybean oil	0.75	1.30	3.24	-	-	-	-	-
Minerals and vitamins	1.57	1.71	2.78	2.14	2.14	1.95	1.89	1.81
Others	0.317	0.387	0.025	-	-	-	-	-

[1] The phases are defined in Table S3.

Table S3. Post-weaning nursery (N1 to N3) and finisher (F1 to F5) phases.

Infection phase	Trait	Post-weaning phase							
		N1	N2	N3	F1	F2	F3	F4	F5
Negative	Weight in[1]	6.26	7.00	11.57	24.95	40.82	58.97	81.65	104.33
	Days	6	15	24	21	21	25	25	31
	Weight out[1]	7.00	11.57	24.95	40.82	58.97	81.65	104.33	129.50
	Feed intake[1]	0.67	5.39	19.03	29.27	40.40	58.77	65.95	84.93
Positive epidemic	Weight in	5.85	7.00	11.57	24.95	40.82	58.97	81.65	104.33
	Days	8	14	25	21	22	25	26	30
	Weight out	7.00	11.57	24.95	40.82	58.97	81.65	104.33	128.09
	Feed intake	1.05	5.39	20.28	29.72	42.54	58.51	67.76	81.57
Positive endemic	Weight in	6.30	7.00	11.57	24.95	40.82	58.97	81.65	104.33
	Days	5	15	25	22	21	25	26	32
	Weight out	7.00	11.57	24.95	40.82	58.97	81.65	104.33	129.68
	Feed intake	0.61	5.38	20.14	31.55	41.32	59.50	69.11	88.22

[1] Weights and feed intake in kg
