# Supplementary material for: The effects of PRRS on the environmental impact of pig production: a life cycle assessment study
Source: Front Vet Sci. 2025 Dec 11;12:1625581. doi: 10.3389/fvets.2025.1625581 (PMC12739953; doi:10.3389/fvets.2025.1625581)
Supplement: Supplementary file 2 [file Data_Sheet_2.pdf]

Data Sheet 2. Results of the comparisons of PRRS-negative farms to the industry average and to PRRS-positive farms.

Table S4. Estimated impacts of PRRS-negative farms as proportions of the North American industry average and of PRRS-positive farms under the ReCiPe-2016 framework. Impact categories marked with ✓ are among the 14 categories shared by ReCiPe and PEF. Entries in **bold text** are significantly (P < 0.05) different from 1.

**Framework ReCiPe-2016**		PRRS-negative / industry average						PRRS-negative / PRRS-positive
Impact category		ReCiPe-Egalitarian		ReCiPe-Hierarchist		ReCiPe-Individualist		ReCiPe-Hierarchist
		cutoff	APOS	cutoff	APOS	cutoff	APOS	cutoff
Fine particulate matter	✓	**0.950**	**0.948**	**0.948**	**0.948**	**0.938**	**0.938**	**0.877**
Fossil resource scarcity	✓	**0.949**	**0.949**	**0.949**	**0.949**	**0.949**	**0.949**	**0.874**
Freshwater ecotoxicity	✓	**0.961**	0.960	**0.960**	**0.960**	0.960	0.960	**0.904**
Freshwater eutrophication	✓	**0.959**	**0.959**	**0.959**	**0.959**	**0.959**	**0.959**	**0.893**
Global warming	✓	**0.951**	**0.946**	**0.950**	**0.950**	**0.949**	**0.949**	**0.876**
Carcinogenic toxicity	✓	0.947	0.947	0.950	0.950	**0.952**	0.952	**0.886**
Non-carcinogenic toxicity	✓	**0.958**	0.958	0.962	0.961	**0.959**	0.959	**0.899**
Ionizing radiation	✓	0.944	0.944	0.930	**0.930**	0.928	0.928	**0.822**
Land use	✓	**0.961**	**0.961**	**0.961**	**0.961**	**0.961**	**0.961**	**0.900**
Marine ecotoxicity		0.957	0.957	**0.959**	**0.959**	0.959	0.959	0.904
Marine eutrophication	✓	**0.963**	**0.963**	**0.963**	**0.963**	**0.963**	**0.963**	**0.899**
Mineral resource scarcity	✓	**0.951**	**0.951**	**0.951**	**0.951**	0.951	**0.951**	**0.884**
Ozone formation, human health		**0.957**	**0.954**	**0.954**	**0.954**	**0.954**	**0.954**	0.888
Ozone formation, terrestrial ecosystems		**0.957**	**0.954**	**0.954**	**0.954**	**0.954**	**0.954**	0.888
Ozone depletion	✓	**0.962**	**0.961**	**0.961**	**0.961**	**0.961**	**0.961**	**0.892**
Terrestrial acidification	✓	**0.95**	**0.949**	**0.949**	**0.949**	**0.949**	**0.949**	**0.879**
Terrestrial ecotoxicity		0.959	0.959	0.959	0.959	0.960	**0.959**	0.904
Water consumption	✓	0.956	0.952	**0.952**	0.952	0.952	**0.952**	**0.876**

Table S5. Estimated impacts of PRRS-negative farms as proportions of the North American industry average and of PRRS-positive farms under the PEF-3.1 framework. Impact categories marked with ✓ are among the 14 categories shared by ReCiPe and PEF. Entries in **bold text** are significantly ($P < 0.05$) different from 1.

**Framework PEF-3.1**		PRRS-negative / industry average		PRRS-negative / PRRS-positive
Impact category		cutoff	APOS	cutoff
Acidification	✓	**0.949**	**0.949**	**0.880**
Carcinogenic toxicity	✓	**0.961**	**0.961**	**0.876**
Carcinogenic toxicity, inorganics		**0.962**	**0.962**	0.897
Carcinogenic toxicity, organics		0.954	0.954	0.873
Fine particulate matter	✓	**0.953**	**0.953**	0.899
Fossil fuel resources	✓	**0.959**	**0.959**	**0.898**
Freshwater ecotoxicity	✓	**0.949**	**0.949**	0.878
Freshwater ecotoxicity, inorganics		0.963	0.963	0.899
Freshwater ecotoxicity, organics		**0.962**	**0.962**	**0.893**
Freshwater eutrophication	✓	**0.953**	**0.953**	**0.885**
Global warming (GWP100)	✓	**0.949**	**0.949**	0.881
Global warming (GWP100), biogenic		**0.950**	**0.950**	**0.893**
Global warming (GWP100), fossil		**0.949**	**0.949**	0.898
Global warming (GWP100), LULUC		**0.960**	**0.960**	0.884
Ionizing radiation	✓	**0.952**	**0.952**	**0.897**
Land use	✓	**0.954**	**0.954**	0.899
Marine eutrophication	✓	**0.950**	**0.950**	0.879
Mineral resources	✓	**0.959**	**0.960**	**0.831**
Non-carcinogenic toxicity	✓	0.933	0.933	**0.868**
Non-carcinogenic toxicity, inorganics		**0.940**	**0.940**	**0.886**
Non-carcinogenic toxicity, organics		0.954	0.953	**0.884**
Ozone depletion	✓	**0.961**	**0.961**	0.889
Photochemical oxidant formation		**0.937**	**0.937**	**0.858**
Terrestrial eutrophication		0.958	0.958	**0.906**
Water use	✓	0.958	0.958	**0.874**

Table S6. Estimated impacts of PRRS-negative farms as proportions of the North American industry average and of PRRS-positive farms under the IPCC-2021 framework. The impact category marked with ✓ is one of the 14 categories shared by ReCiPe and PEF. All entries are significantly (P < 0.05) different from 1.

**Framework IPCC-2021**		PRRS-negative / industry average		PRRS-negative / PRRS-positive
Impact category		cutoff	APOS	cutoff
AGTP-100		**0.942**	**0.942**	**0.856**
AGTP-50		**0.942**	**0.943**	**0.859**
AGWP-100		**0.950**	**0.949**	**0.875**
AGWP-20		**0.949**	**0.949**	**0.878**
AGWP-500		**0.946**	**0.947**	**0.870**
CGTP-100		**0.948**	**0.948**	**0.887**
CGTP-50		**0.948**	**0.948**	**0.887**
GTP-100		**0.949**	**0.949**	**0.873**
GTP-50		**0.949**	**0.950**	**0.874**
GWP-100	✓	**0.950**	**0.949**	**0.875**
GWP-20		**0.948**	**0.949**	**0.878**
GWP-500		**0.947**	**0.947**	**0.870**

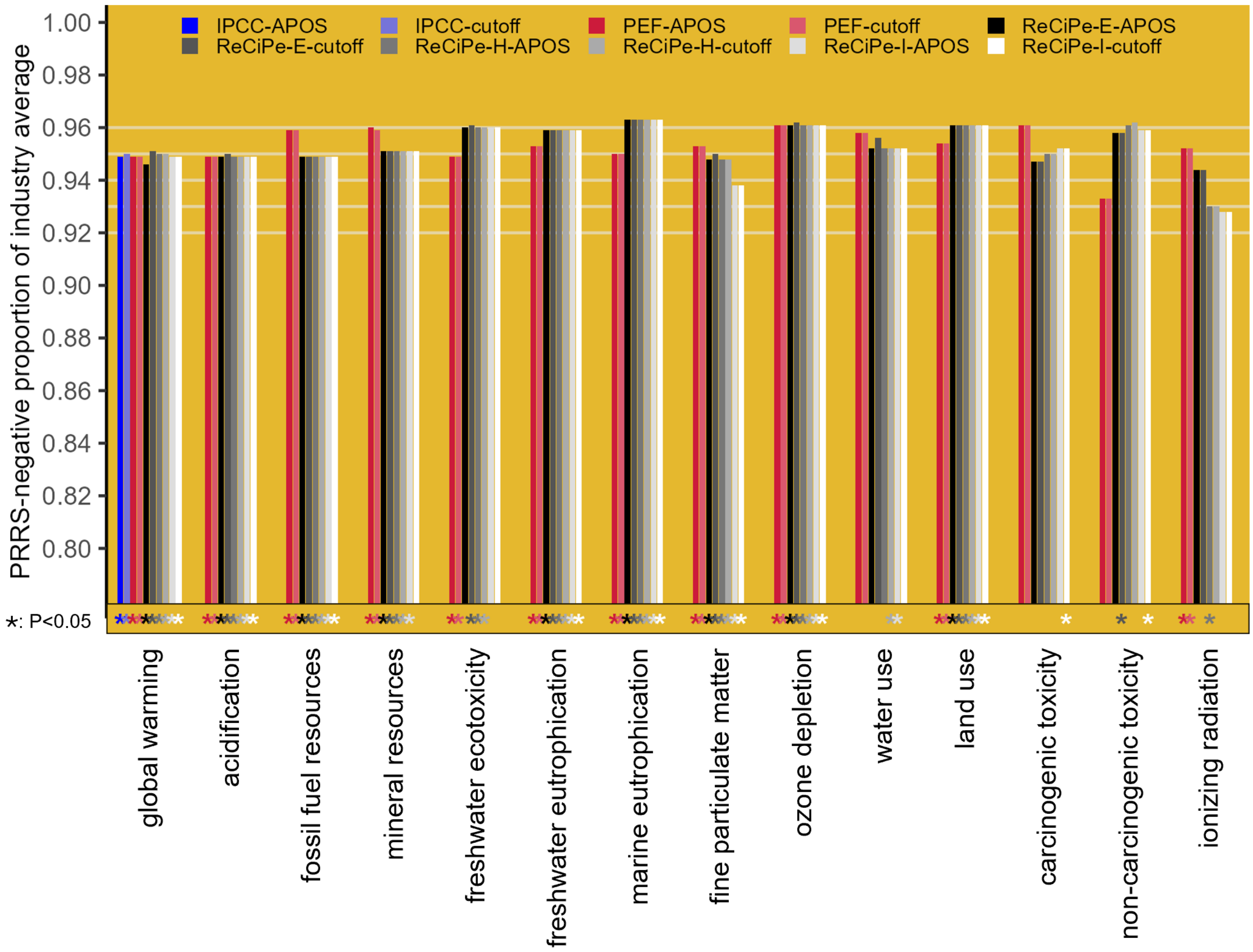

Figure B1. Performance of North American pig production systems in the *Negative* PRRSV infection phase as compared to the North American industry average, for 14 environmental impact categories, as modeled in ten LCIA frameworks (ReCiPe-2016 Egalitarian, Hierarchist and Individualist, cutoff and APOS; PEF-3.1 cutoff and APOS; IPCC-2021 cutoff and APOS). The IPCC cases for global warming (blue bars) are IPCC's GWP-100 category; IPCC does not consider the other impact categories. Lower values are more favorable; the asterisks below the bars indicate cases where the estimate for PRRS-negative systems is significantly ($P<0.05$) different from 1 (i.e. from the industry average). The scaling of the Y-axis is the same as in Figure B2.

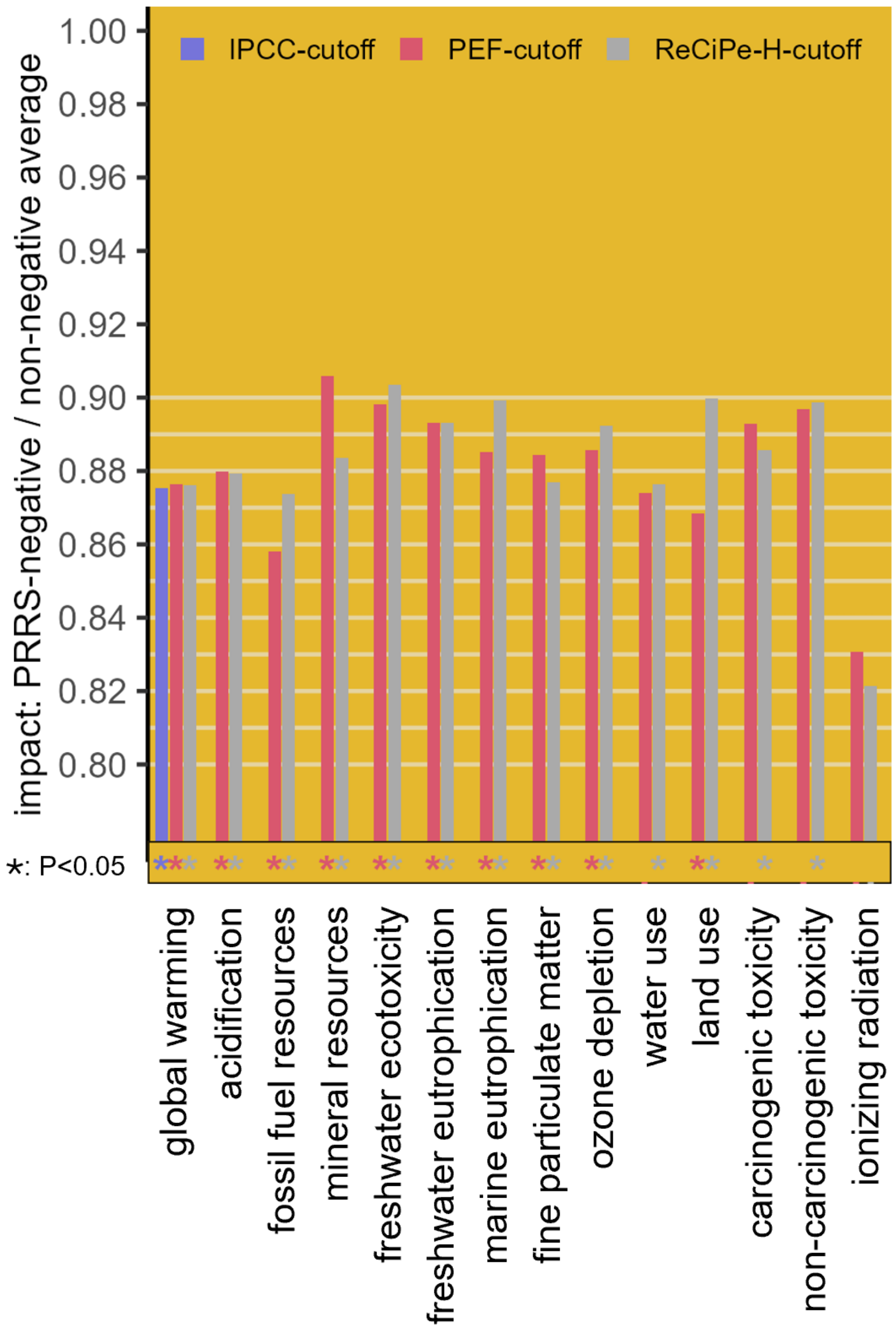

Figure B2. Performance of North American pig production systems in the PRRSV-negative infection phase as compared to PRRSV-positive ones, for 14 environmental impact categories, as modeled in three LCIA frameworks for the Ecoinvent cutoff system model (ReCiPe-2016 Hierarchist, PEF-3.1 and IPCC-2021). Lower values are more favorable; the asterisks below the bars indicate cases where the estimate for PRRS-negative systems is significantly ($P<0.05$) different from 1 (i.e. from PRRS-epidemic systems). The color coding, further formatting, and scaling of the Y-axis are the same as in Figure B1.
